# Graphene Aerogel Growth on Functionalized Carbon Fibers

**DOI:** 10.3390/molecules25061295

**Published:** 2020-03-12

**Authors:** Katerina Vrettos, Konstantinos Spyrou, Vasilios Georgakilas

**Affiliations:** 1 Department of Material Science, University of Patras, 26504 Patras, Greece; c.vrettos0@gmail.com; 2 Department of Materials Science and Engineering, University of Ioannina, 45110 Ioannina, Greece; konstantinos.spyrou1@gmail.com

**Keywords:** carbon fibers, surface treatment, grafting, graphene aerogel

## Abstract

Graphene aerogel (GA) is a lightweight, porous, environmentally friendly, 3D structured material with interesting properties, such as electrical conductivity, a high surface area, and chemical stability, which make it a powerful tool in energy storage, sensing, catalyst support, or environmental applications. However, the poor mechanical stability that often characterizes graphene aerogels is a serious obstacle for their use in such applications. Therefore, we report here the successful mechanical reinforcement of GA with carbon fibers (CFs) by combining reduced graphene oxide (rGO) and CFs in a composite material. The surfaces of the CFs were first successfully desized and enriched with epoxy groups using epichloridrine. Epoxy-functionalized CFs (epoxy-CFs) were further covered by reduced graphene oxide (rGO) nanosheets, using triethylene tetramine (TETA) as a linker. The rGO-covered CFs were finally incorporated into the GA, affording a stiff monolithic aerogel composite. The as-prepared epoxy-CF-reinforced GA was characterized by spectroscopic and microscopic techniques and showed enhanced electrical conductivity and compressive strength. The improved electrical and mechanical properties of the GA-CFs composite could be used, among other things, as electrode material or strain sensor applications.

## 1. Introduction

Graphene oxide (GO) is the most common graphene derivative [1]. It is formed by the oxidative treatment of graphite and is characterized by a large amount of oxygen groups, which are spread over the graphenic surface. Carboxylates mainly at the edges, epoxy, and hydroxylates at the core provide a strong hydrophilic character and dramatically reduce the aromatic character of GO. The treatment of GO with reducing agents leads to the partial removal of the oxygen groups and the reconstruction of aromaticity, to the so-called reduced GO (rGO). Depending on the reductive treatment, rGO shows often remarkable electrical conductivity, comparable with pristine graphene. On the other hand, the removal of the majority of the oxygen groups results in the decrease in the hydrophilic character of rGO and the formation of aggregates when they are dispersed in water [2].

Hydrothermal reductive treatment of GO often leads to the formation of a stable rGO hydrogel, depending on the conditions. GO is a highly hydrophilic 2D material, which, under reductive conditions, is partially gaining its aromaticity and self-assembled in a 3D structure due to *π-π* stacking interactions. The oxygen groups on the graphene surface, as well as the entrapped hydrophilic reducing agent, are often responsible for the entrapment of a large amount water between the rGO nanosheets, leading to the formation of stable hydrogels, which can be transformed into a graphene aerogel (GA), after water removal by a freeze-drying procedure.

GA is a lightweight, porous, environmentally friendly 3D structured material, with electrical conductivity, chemical inertness and a high surface area [3,4,5,6]. It could be used in several applications, including in supercapacitors, lithium ion batteries, fuel or solar cells, and for environmental purposes such as water purification, gas separation, or electromagnetic interference shielding. The use of GA in most applications could be largely promoted by a significant mechanical reinforcement, which is a real challenge, taking into consideration the poor mechanical properties of GA [3,4,5,6].

On the other hand, carbon fibers (CFs) are a graphitic material that have been used widely for the reinforcement of polymers, due to their remarkable mechanical, thermal, and electrical properties as well. CFs/polymer composites have been used in applications such as in aerospace, automotive, nuclear engineering, where strength, stiffness, and lightweight materials are critical requirements [7,8,9,10,11]. However, their smooth and inert graphitic surface, almost free of functional groups, does not favor the adhesion with matrix polymer molecules, weakening the load that is transferred from the matrix to the CFs, and limiting the mechanical reinforcement [8,9]. Therefore, in the past decades, many research efforts have been made to modify chemically the surface of CFs, and thus to improve the interfacial adhesion in the resulting composites. However, there has been limited success up to now in the sufficient functionalization of the CF’s surface. In most cases, the CF’s surface is treated with strong acids, such as a nitric or nitric/sulfuric acid mixture, to introduce a limited amount of oxygen groups (mainly carboxylates) [12,13,14]. Carboxylates are then used as reactive sites to graft on the CF’s surface coupling agents such as simple diamines, dialdehydes [15,16], or, more specifically, polyphosphazenes and siloxanes [17,18,19]. The decoration of CFs with coupling agents increases the wettability and chemical reactivity of CFs, and this improves the adhesion between the CFs and polymer matrices. Recently, GO has been also used to modify the CF’s surface, improving the interfacial properties of the composite material [20]. Furthermore, efforts have appeared in the literature that combined CFs and GA in a 3D composite structure, ideally having the properties of both components [21,22].

In the present article, we describe an effective enrichment of CFs surface with epoxy groups, and their successful incorporation in a GA, forming a 3D structured monolithic aerogel composite with improved electrical and mechanical properties. The CFs were first desized and then functionalized with epoxy groups to enhance the binding sites of the CFs, using, for the first time, epichlorohydrin. Epoxy-functionalized CFs (epoxy-CFs) were then covered by rGO nanosheets and finally incorporated successfully into the GA, during a sol-gel hydrothermal reduction process of GO. The as-prepared CFs/rGO monolithic aerogel composite was fully characterized by spectroscopic and microscopic techniques, and finally showed enhanced electrical conductivity and compression stability. Due to these characteristics, the CFs/rGO aerogel composite could be used, among other things, as an electrode material or in strain sensor applications [23].

## 2. Results and Discussion

**Figure 1:**
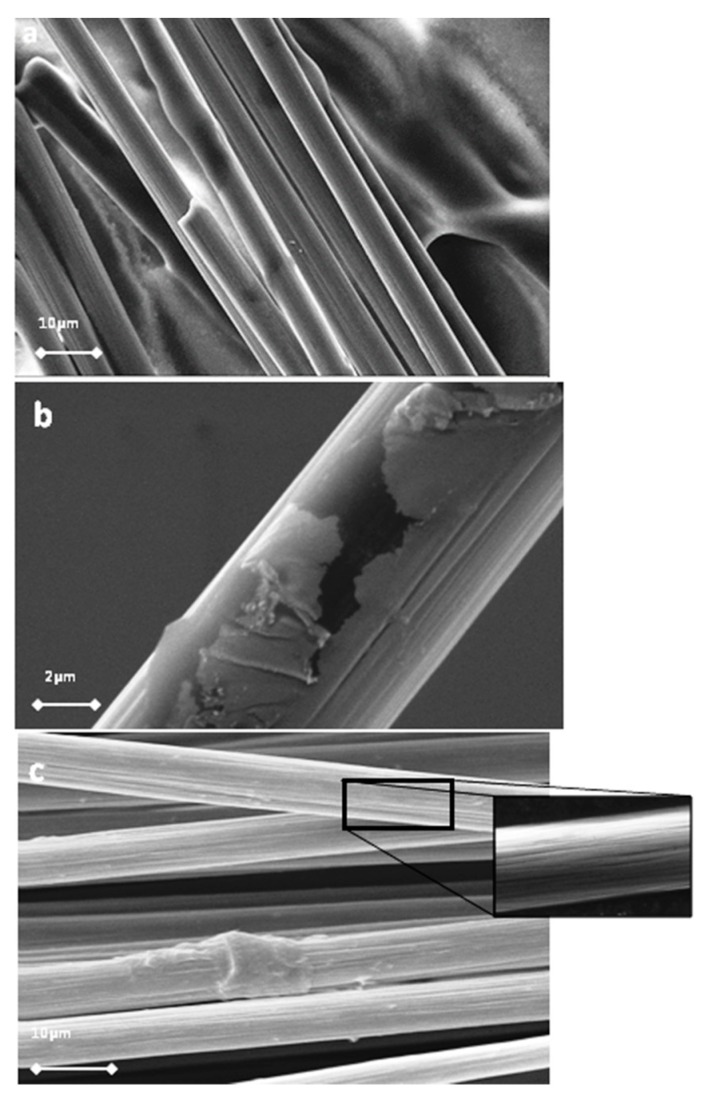
Scanning electron microscopy (SEM) images of carbon fibers (CFs) before (**a**) and after the treatment with acetone (**b**,**c**).

Initially, the CFs were desized in acetone [24] and then oxidized using an acidic treatment under ultrasonication. After this procedure, a plethora of active oxygen groups, mainly hydroxylates and carboxylates, appeared on the surface of the CFs, increasing the total surface energy and polarity, which was helpful to improve the wettability of the CFs [25,26]. The morphology of the CFs was studied using scanning electron microscopy (SEM), and characteristic micrographs are given in Figure 1.

**Figure 2:**
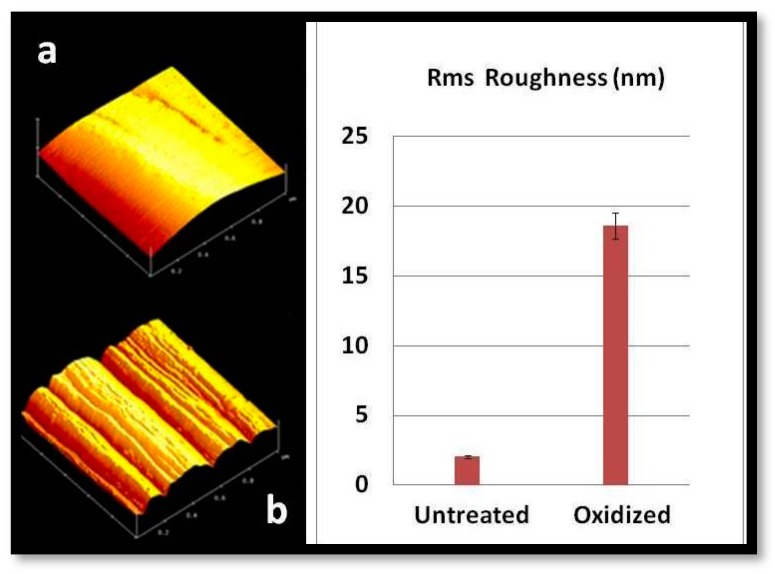
Atomic force microscopy (AFM) topography images of (**a**) untreated and (**b**) oxidized CFs.

The polymer removal alters the morphology and increases the roughness of the CFs’ surface. The characteristic change in the roughness of CFs after desizing is indicated by atomic force microscopy (AFM) images, as shown in Figure 2. The surfaces of untreated CFs seem to be relatively neat and smooth. Few narrow grooves, distributed in parallel along the longitudinal direction of the fiber, are due to the fiber manufacture process. Compared with the untreated CFs (Figure 1a), the fiber surface becomes rougher after oxidative treatment.

**Figure 3:**
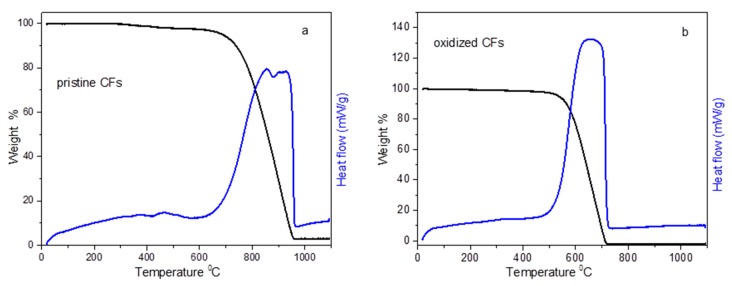
Thermo gravimetric analysis (TGA) and heat flow graphs of pristine (**a**) and oxidized CFs (**b**).

Thermogravimetric and differential thermal analysis (TG-DTA) curves of pristine CFs and oxidized CFs are depicted in Figure 3. Pristine CFs were stable until 700 °C in the air, while, after desizing and acid treatment, oxidized CFs were decomposed much easier between 500 and 700 °C (see Figure 3). The decomposition of both the pristine and oxidized CFs was accompanied by an analogous exothermic peak, recorded in DTA as expected. Pristine CFs also indicated a 2% weight loss between 350 and 500 °C, which could be attributed to the removal of the sizing agent.

**Figure 4:**
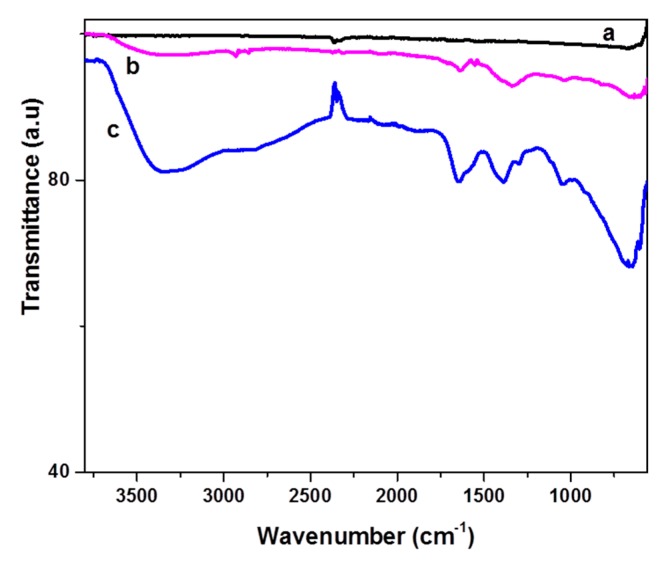
Fourier–transform infrared spectrometer (FT-IR) spectrum of (**a**) pristine CFs (**b**) acid treated CFs and (**c**) epoxy-functionalized CFs (epoxy-CFs).

The Fourier–transform infrared spectrometer (FT-IR) spectrum of the oxidized CFs (see line b in Figure 4) showed a few weak but characteristic peaks due to the presence of oxygen groups on their surface, in contrast with the featureless spectrum of pristine CFs (see line a in Figure 4). The peaks at 3300 (OH stretching vibrations) and 1033 cm^−1^ (C-O stretching vibrations) indicated the appearance of hydroxyl groups on the CF surface. The peaks at 1715 and 1640 cm^−1^ (C=O stretching) could be attributed to the presence of carboxylates and carbonyl groups, respectively, while peaks at 2860, 2930, and 1335 cm^−1^ indicate sp^3^ C-H stretching [27,28,29].

**Figure 5:**
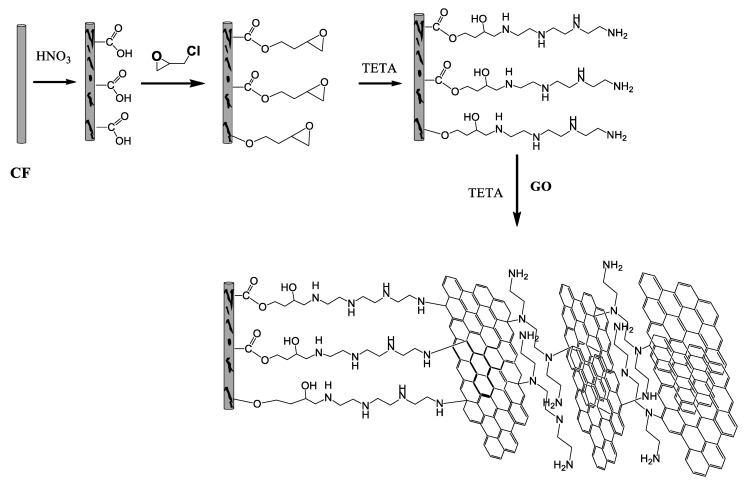
Schematic representation of the formation of CFs/ reduced GO (rGO) composite aerogel.

Epichlorohydrin was then grafted on the CFs following mainly two different pathways. It can be added to the carboxylates or OH groups using the epoxy or chlorine end, respectively, as shown in Figure 5. Both pathways in the alkaline environment lead to the formation of epoxy groups on the surface of the CFs (epoxy-CFs). The FT-IR spectrum of the epoxy-CFs showed their enrichment with hydroxyl and epoxy groups, as indicated by the OH stretching vibration at 3330 cm^−1^ and C-O-C, epoxy stretching vibration at 1290 and 1050 cm^−1^. The characteristic CH and C=C vibrations at 2850 (stretching), 1400 (in plane bending), and 1647 cm^−1^ (stretching), respectively, also appeared (see line c in Figure 4).

**Figure 6:**
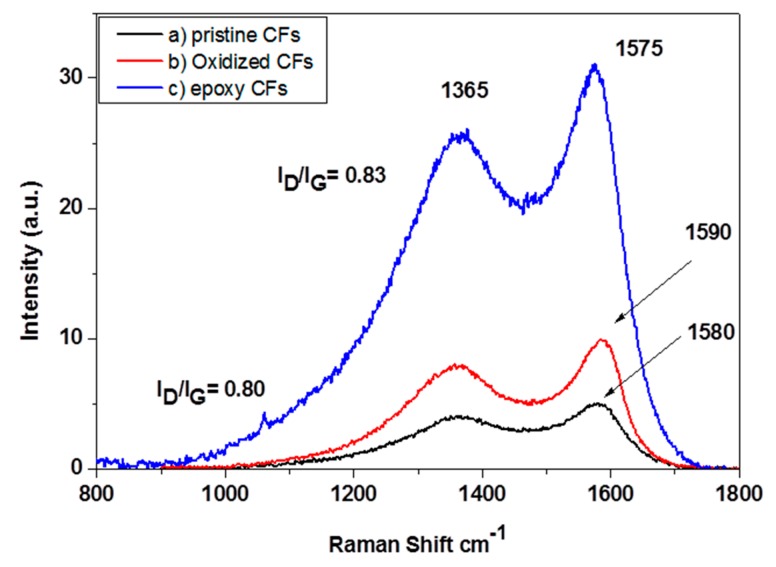
Raman spectra of (**a**) pristine (**b**) oxidized and (**c**) epoxy-CFs.

The Raman spectra of pristine, oxidized and epoxy-CFs are shown in Figure 6. The spectrum of the CFs contains two characteristic peaks, which are assigned to the graphitic E_2g_ G mode at ~1580 cm^−1^ and the disorder D mode at ~1365 cm^−1^. In the spectrum of epoxy-CFs, the G band appeared slightly shifted to 1590 cm^−1^ due to the contribution of the D’ band at around 1610 cm^−1^, which is more intense after the introduction of oxygen groups on the CFs’ surface. The I_D_/I_G_ ratio after the reaction with epichlorohydrin was slightly increased due to the introduction of epoxy groups [30].

**Figure 7:**
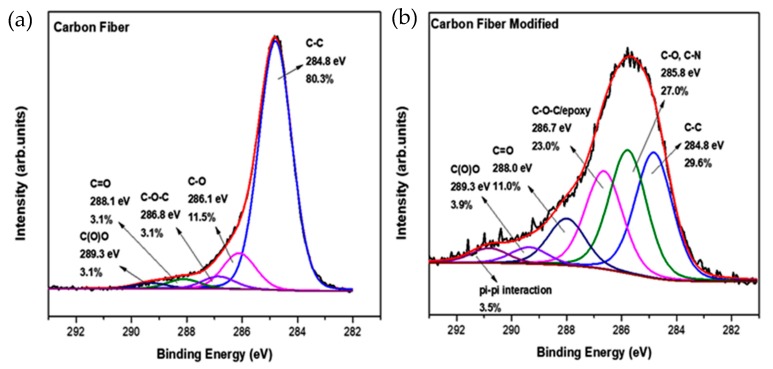
C1s photoelectron peak of (**a**) pristine CFs and (**b**) epoxy-CFs.

In order to identify further the functional chemical groups of the epoxy-CFs, X-ray photoelectron spectroscopy (XPS) measurements were employed. From the C1s high resolution photoelectron spectra (Figure 7), several changes after the chemical modification were deduced. The most important information that was collected here was the reduction in the C-C frame from 80.3% for the pristine CFs to 29.6% for the epoxy-CFs. The peak at 286.1 eV is increased because of the addition of C-O and also C-N bonds. The existence of these two functionalities explains the small shift from 286.1 eV, due to CFs, to 285.8 eV. Important evidence for the successful functionalization of CFs is the significant increase in the photoelectron peak at 286.8 eV from 3.1% to 23.0%. This increase is due to the epoxy group created after the chemical functionalization [29,31].

**Figure 8:**
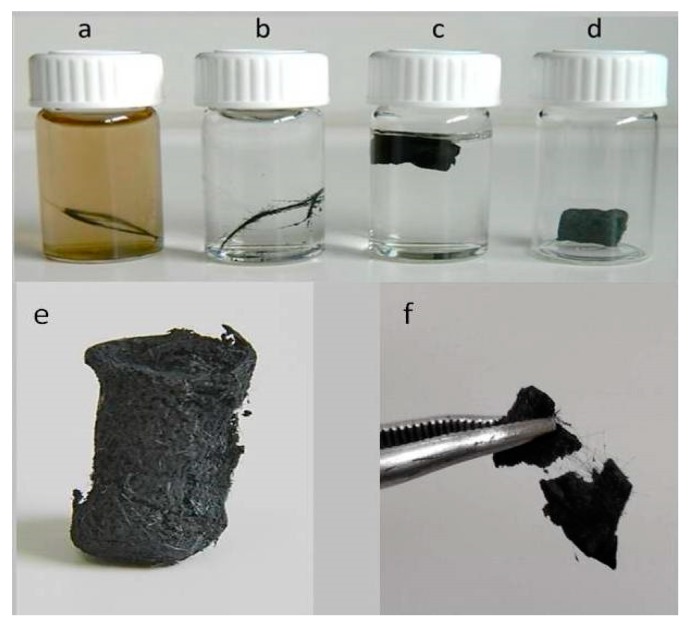
Photo of (**a**) epoxy-CFs in an alkaline solution of GO, (**b**) epoxy-CFs covered by the rGO nanosheets after hydrothermal heating, (**c**) CFs/rGO hydrogel and (**d**,**e**) aerogel, (**f**) CFs/rGO aerogel separated in two pieces using forcepts, where CFs are revealed from the internal.

Epoxy-CFs were transferred to a diluted GO dispersion in an alkaline solution of triethylene tetramine (TETA) and heated hydrothermally at 100 °C in a sealed bottle. At this stage, the brown GO solution becomes colorless after the reaction, indicating the successful immobilization of the rGO nanosheets on the external surface of the CFs (see Figure 8a,b). The rGO-covered epoxy-CFs finally underwent a second hydrothermal treatment with a larger amount of GO dispersed in alkaline solution of TETA. Under these conditions, the GO was reduced partially and aggregated, forming a stable hydrogel (see Figure 8c). Through the nucleophilic addition to the epoxy ring opening functionalization, TETA acts here as a reducing and coupling agent on both CFs and rGO nanosheets, and thus contributes significantly to the hydrogel formation as a bridge molecule. The insertion of epoxy-CFs between the rGO nanosheets resulted in the successful incorporation of the former in the hydrogel (see Figure 8c). Finally, after freeze drying, the CFs-supported GA (CFs/rGO aerogel) was formed (see Figure 8d).

**Figure 9:**
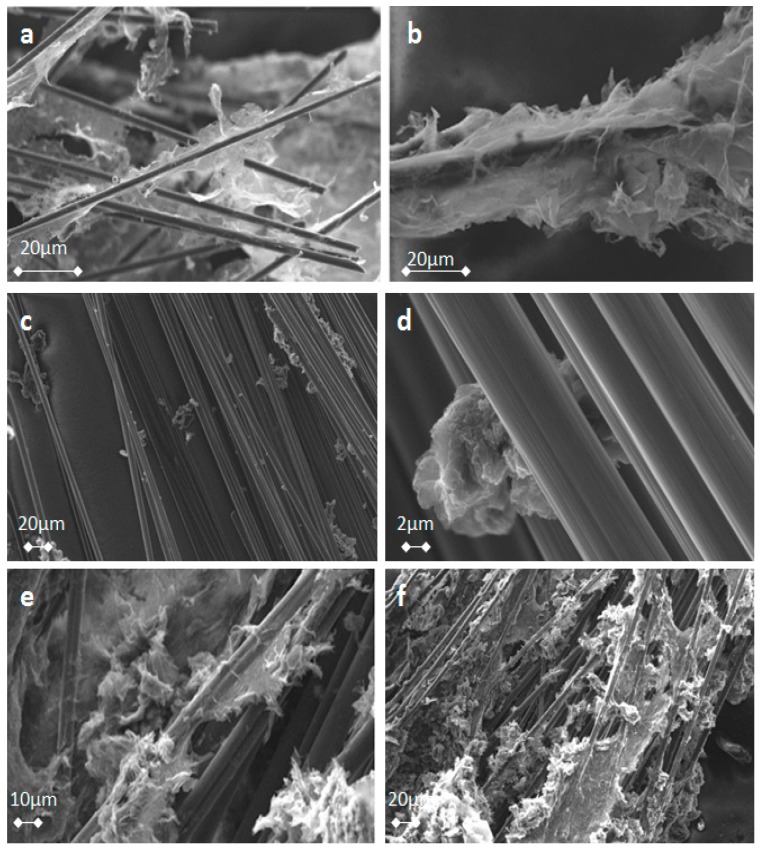
SEM images of epoxy-CFs (**a**,**b**) and pristine CFs (**c**,**d**) covered by rGO nanosheets, and CFs/rGO aerogel (**e**,**f**).

CFs have a mean size of 0.3–0.5 cm and were randomly oriented in the GA, as observed optically under a microscope. This is a consequence of the random dispersion of CFs into the mixture of the GO before the hydrothermal treatment. CFs/rGO aerogel can be also formed in a single stage hydrothermal procedure by directly dispersing epoxy-CFs in a concentrated dispersion of GO in alkaline solution of TETA. In Figure 9, several characteristic images of rGO-covered epoxy-CFs are presented (Figure 9a,b,e,f), in comparison with poorly rGO-covered pristine CFs (Figure 9c,d) that were formed when pristine CFs were used instead of epoxy-CFs. As shown in Figure 9a–d, rGO nanosheets have extensively covered the surface of the epoxy-CFs, while the absence of epoxy groups in pristine CFs leads to much less coverage by rGO, as shown in Figure 9c,d. Finally, Figure 9e,f indicate the successful incorporation of CFs in the CFs/rGO aerogel. Due to the interaction between the epoxy-CFs and GO in the presence of the TETA bridge molecules, the epoxy-CFs reinforced GA were more condensed, having a lower volume (0.6 cm^3^) than the pure rGO aerogel (0.9 cm^3^). In addition, taking into consideration the masses of the components and the final products, epoxy-CFs reinforced GA showed a higher density (31.6 mg/cm^3^) in comparison to pure rGO aerogel (12,5 mg/cm^3^), due to the lower volume and the presence of CFs. The mass fraction of CFs in the CFs/rGO aerogel was estimated to be 0.31 and the volume fraction to be 5 × 10^−3^.

### 2.1. Electrical Conductivity

**Figure 10:**
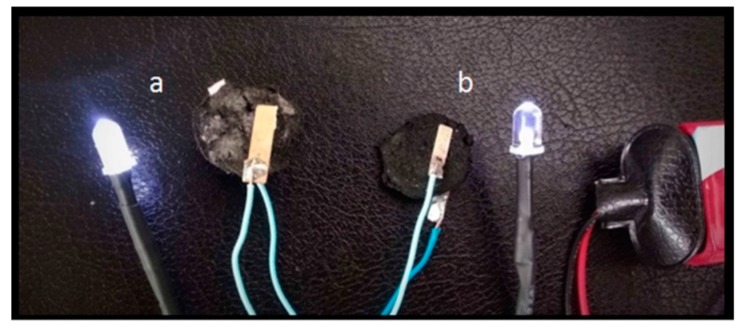
Electrical circuits with (**a**) CFs/rGO and (**b**) rGO aerogel monoliths. The intense light of the LED lamp in circuit (**a**) indicates the increased conductivity of CFs/rGO aerogel.

It is known that GAs are electrically conductive, due to the recovered aromaticity after the reduction of GO. CFs are also conductive, and thus the final CFs/rGO composite is highly conductive as expected (see Figure 10). In fact, the resistivity of a CFs/rGO aerogel monolith was measured to 28.8 Ω m, while an analogous rGO aerogel monolith was measured to 129.6 Ω m (see Table 1). The samples had a cylindrical shape and the resistance was measured by adapting two electrodes at the upper and lower surface of the cylinders (see experimental part). It is important to note here that the orientation of the CFs in the aerogel was random and not involved with the increased conductivity of the epoxy-CFs supported GA.

### 2.2. Mechanical Reinforcement

**Figure 11:**
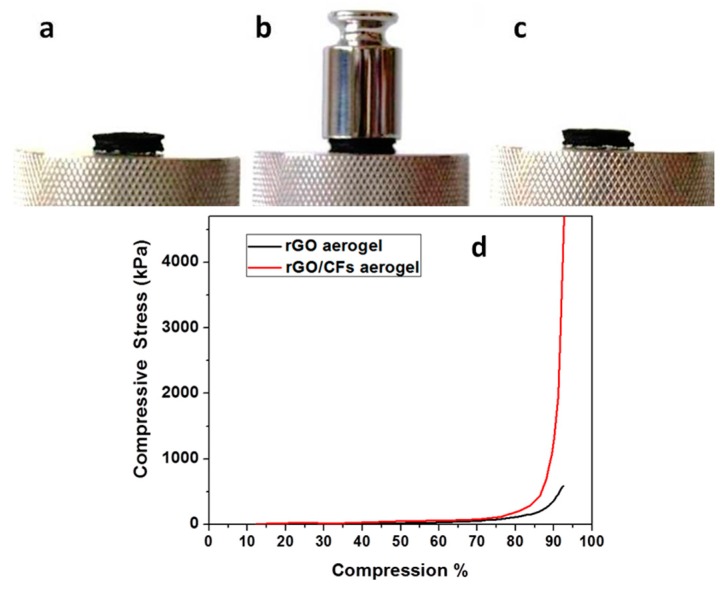
(Upper) CFs/rGO cylindrical aerogel monolith before (**a**) during (**b**) and after (**c**) the compression, with a 50 g standard weight. (**d**) The stress/compression diagram of rGO and CFs/rGO aerogel monoliths.

In a recent previous article, we showed that TETA-promoted rGO aerogels can be compressed to about 50% of the initial thickness by the placement of a 50 g standard weight on a GA cylindrical monolith, while the rGO aerogels promoted by aromatic diamines were compressed almost elastically [32]. Here, we demonstrate that TETA-promoted GA reinforced with CFs was not compressed under the same conditions, indicating the remarkable role of CFs on the mechanical reinforcement of the CFs/rGO aerogel (see Figure 11a–c). In contrast, GA reinforced with pristine CFs by the same procedure is fragile and mechanically very unstable, leading to negative results as regards mechanical measurements. This fact indicated the crucial role of epoxy groups in the successful incorporation of CFs in the GA.

A similar conclusion was drawn by comparing the diagrams of compressive stress to compression of the rGO and the CFs/rGO aerogels (see Figure 11d). In fact, the rGO aerogel was compressed to about 90% with a 400 kPa stress, while in the case of the CFs/rGO aerogel, a remarkably higher compressive stress—about 1300 kPa—was applied to achieve a similar compression. This 3D porous, conductive, and highly stable CFs/rGO structure could become a highly promising material for applications in lightweight conductive cables, energy storage, catalysts, and functional textiles. The unique structure of those materials paves the way to design and fabricate lightweight porous materials with high performance.

## 3. Materials and Methods

### 3.1. Materials

Commercially available CFs, T700SC (Fiber Max, Volos, Greece) were used in this work. Epichlorohydrin (Alfa Aesar, Kandel, Germany), sodium hydroxide (NaOH, Sigma-Aldrich, St Louis city, MO, USA), ammonium hydroxide (NH_3_, CARLO ERBA Reagents S.A.S., Barcelona, Spain), TETA (Sigma-Aldrich, St Louis city, MO, USA), nitric acid (HNO_3_, 65%, CARLO ERBA Reagents S.A.S., Barcelona, Spain), and powder graphite (Sigma-Aldrich, St Louis city, MO, USA) were used without further treatment. GO was prepared in the lab, according to Staudenmaier’s method [33] and the synthesis is described in detail, in ref [34].

### 3.2. Characterization

TGA/DSC measurements were performed on pristine and oxidized CFs (~10 mg) by means of a SETARAM SETSYS Evolution 18 Analyzer (SETARAM Instrumentation, Caluire, France) with Al_2_O_3_ crucibles, in the range of 25–1100 °C. A heating rate of 10 °C/min under air flow (16 mL/min) while used and purging was applied well before initiating the heating ramp. Buoyancy corrections were carried out through blank measurements.

Scanning electron microscopy (SEM) was carried out on a Zeiss EVO-MA10 (Carl Zeiss Microscopy GmbH, Jena, Germany). Infrared spectra were measured on a Fourier–transform infrared spectrometer (FT-IR) using the ATR technique on an IRTracer-100 Shimadzu spectrometer (Shimadzu Europa GmbH, Duisburg, Germany). Raman spectra were collected with a Raman system Lab-Ram HR Evolution RM (Horiba-Scientific, Kyoto, Japan) using a laser excitation line at 532 nm (laser diode). The laser power was 1.082 mV. All Raman parameters have been carefully controlled to avoid changes in the graphene materials. Bulk resistance was measured using a Keithley 2401 multimeter (Keithley Instruments, Solon, OH, USA), using two indium tin oxide (ITO) glass slides as electrodes that covered the upper and lower surface of the samples. Atomic force microscopy (AFM) measurements were performed in tapping mode with a multimode Nanoscope IIIa (Bruker, Billerica, MA, USA), using RTESPA-300 silicon cantilevers with a nominal tip radius 8 nm. The values of the stress/compression diagrams of rGO and rGO/CFs aerogel monoliths were recorded with a Hounsfield H20K-W test machine (rate 1.5 mm/min, Hounsfield Test Equipment, Red Hill, England).

X-ray photoelectron spectroscopy (XPS) measurements were performed in an ultra-high vacuum at a base pressure of 2 × 10^−10^ mbar, with a SPECS GmbH spectrometer (SPECS Surface Nano Analysis GmbH, Berlin, Germany) equipped with a monochromatic MgKa source (hv = 1253.6 eV), and a Phoibos-100 hemispherical analyzer (SPECS Surface Nano Analysis GmbH, Berlin, Germany). The spectra were collected in normal emission and the energy resolution was set to 1.16 eV to minimize measuring time. All binding energies were referenced to the C1s core level at 284.8 eV. Spectral analysis included a Shirley background subtraction and a peak deconvolution employing mixed Gaussian-Lorentzian functions, in a least square curve-fitting program (WinSpec) developed at the Laboratoire Interdisciplinaire de Spectroscopie Electronique, University of Namur, Belgium.

### 3.3. Oxidation Treatment and Functionalization of CFs

Some 100 mg of CFs were heated in acetone for 48 h at 60 °C. After the drying of the desized CFs, they were oxidized in conc. HNO_3_ at 100 °C for 2 h in a sonication bath. Subsequently, the oxidized CFs were washed several times with deionized water until reaching pH ~ 7 and dried at 100 °C under vacuum. Oxidized CFs were placed in a solution of epichlorohydrin, while an ethanolic solution of NaOH was added slowly during refluxing (95 °C) for 3 h. The epoxy-CFs were finally washed with acetone and dried under a vacuum.

### 3.4. Formation of CFs/rGO Aerogel

Epoxy-CFs (6 mg) were placed into a concentrated solution of TETA in water (50% *v*/*v*), at 80 °C for 24 h. After a thorough washing, the as-prepared amine functionalized CFs (amino-CFs) were transferred in an alkaline GO dispersion (2 mg of GO, 50 μL conc. NH_3_ in 20 mL H_2_O) at 95 °C for 24 h. The as prepared rGO functionalized CFs were then placed in a dispersion of GO (10 mg) and TETA (10 μL) in 20 mL of water, and the mixture was heated in a sealed bottle at 95 °C for 24 h. The resulting hydrogel was washed several times with water and lyophilized for 24 h.

## 4. Conclusions

In this work, we demonstrated that epoxy-functionalized CFs can be successfully incorporated into GA by grafting rGO to their surface and forming a composite aerogel monolith with improved electrical and mechanical properties, due to the presence of CFs. The role of epichlorohydrin in the introduction of epoxy groups to the CFs surface was crucial, since epoxy groups are the key for the successful incorporation of CFs into rGO aerogel. The as-prepared CFs/rGO aerogel showed at least four times lower electrical resistivity than rGO aerogel, since the desized CFs function as conducting pathways within the porous structure. Despite desizing, the contribution of chemical functionalization to the surface of CFs to the mechanical properties of the final composite was also remarkable.

## Figures and Tables

**Table 1:** Bulk resistance (R) and resistivity (r) of rGO and CFs/rGO aerogels.

	S (m^2^) × 10^−4^	*l* (m)	R (kΩ)	ρ (Ω m)
rGO	1.8	0.005	3.6	129.6
CFs/rGO	1.8	0.005	0.8	28.8

## References

[1] Chen D., Feng H., Li J. (2012). Graphene Oxide: Preparation, Functionalization, and Electrochemical Applications Information. Chem. Rev..

[2] Pei S., Cheng H.M. (2012). The reduction of graphene oxide. Carbon.

[3] Hu H., Zhao Z., Wan W., Gogotsi Y., Qiu J. (2013). Ultralight and Highly Compressible Graphene Aerogels. Adv. Mater..

[4] Tang G., Jiang Z.G., Li X., Zhang H.B., Dasari A., Yu Z.Z. (2014). Three-dimensional graphene aerogels and their electrically conductive composites. Carbon.

[5] Mao J., Iocozzia J., Huang J., Meng K., Lai Y., Lin Z. (2018). Graphene aerogels for efficient energy storage and conversion. Energy Environ. Sci..

[6] Li C., Ding M., Zhang B., Qiao X., Liu C.Y. (2018). Graphene aerogels that withstand extreme compressive stress and strain. Nanoscale.

[7] Prashanth S., Subbaya K.M., Nithin K., Sachhidananda S. (2017). Fiber Reinforced Composites-A Review. J. Mater. Sci. Eng..

[8] Chen J., Xu H., Liu C., Mi L., Shen C. (2018). The effect of double grafted interface layer on the properties of carbon fiber reinforced polyamide 66 composites. Compos. Sci. Technol..

[9] Koutroumanis N., Manikas A.C., Pappas P.N., Petropoulos F., Sygellou L., Tasis D., Papagelis K., Anagnostopoulos G., Galiotis C. (2018). A novel mild method for surface treatment of carbon fibres in epoxy matrix Composites. Compos. Sci. Technol..

[10] Rajak D.K., Pagar D.D., Menezes P.L., Linul E. (2019). Fiber-Reinforced Polymer Composites: Manufacturing, Properties, and Applications. Polymers.

[11] Anguita J.V., Smith C.T.G., Stute T., Funke M., Delkowski M., Silva S.R.P. (2019). Dimensionally and environmentally ultra-stable polymer composites reinforced with carbon fibres. Nat. Mater..

[12] Tiwari S., Bijwe J. (2014). Surface Treatment of Carbon Fibers-A Review. Procedia Technol..

[13] Dai Z., Shi F., Zhang B., Li M., Zhang Z. (2011). Effect of sizing on carbon fiber surface properties and fibers/epoxy interfacial adhesion. Appl. Surf. Sci..

[14] Zhang G., Sun S., Yang D., Dodelet J.P., Sacher E. (2008). The surface analytical characterization of carbon fibers functionalized by H2SO4/HNO3 treatment. Carbon.

[15] Choi M.H., Jeon B.H., Chung I.J. (2000). The effect of coupling agent on electrical and mechanical properties of carbon fiber/phenolic resin composites. Polymer.

[16] Pittman Jr C.U., Wu Z., Jiang W., He G.-R., Wu B., Li W., Gardner S.D. (1997). Reactivities of amine functions grafted to carbon fiber surfaces by tetraethylenepentamine. Designing interfacial bonding. Carbon.

[17] Becker-Staines A., Bremser W., Tröster T. (2019). Poly(dimethylsiloxane) as Interphase in Carbon Fiber-Reinforced Epoxy Resin: Topographical Analysis and Single-Fiber Pull-Out Tests. Ind. Eng. Chem. Res..

[18] Cheng X., He Z., Luo Y., Zhang X., Lei C. (2019). Manipulating Interfacial Strength of Polyphosphazene Functionalized Carbon Fiber Composites. Polym. Compos..

[19] Zhang X., Xu H., Fan X. (2014). Grafting of amine-capped cross-linked polyphosphazenes onto carbon fiber surfaces: A novel coupling agent for fiber reinforced composites. Rsc Adv..

[20] Liu L., Yan F., Li M., Zhang M., Xiao L., Ao Y. (2018). Self-assembly of graphene aerogel on carbon fiber for improvement of interfacial properties with epoxy resin. Mater. Lett..

[21] Gao B., Zhang R., He M., Sun L., Wang C., Liu L., Zhao L., Cui H., Cao A. (2016). Effect of a multiscale reinforcement by carbon fiber surface treatment with graphene oxide/carbon nanotubes on the mechanical properties of reinforced carbon/carbon composites. Compos. Part A Appl. Sci. Manuf..

[22] Keyte J., Pancholi K., Njuguna J. (2019). Recent Developments in Graphene Oxide/Epoxy Carbon Fiber-Reinforced Composites. Front. Mater..

[23] Qin Y., Qu M., Pan Y., Zhang C., Schubert D.W. (2020). Fabrication, characterization and modelling of triple hierarchic PET/CB/TPU composite fibres for strain sensing. Compos. Part A Appl. Sci. Manuf..

[24] Zhao M., Meng L., Ma L., Ma L., Yang X., Huang Y., Ryu J.E., Shankar A., Li T., Yan C. (2018). Layer-by-layer grafting CNTs onto carbon fibers surface for enhancing the interfacial properties of epoxy resin composites. Compos. Sci. Technol..

[25] Peng Q., Li Y., He X., Lv H., Hu P., Shang Y., Wang C., Wang R., Sritharan T., Du S. (2013). Interfacial enhancement of carbon fiber composites by poly(amido amine) functionalization. Compos. Sci. Technol..

[26] Xu H., Zhang X., Liu D., Chun Y., Fan X., Chun (2014). A high efficient method for introducing reactive amines onto carbonfiber surfaces using hexachlorocyclophosphazene as a new coupling agent. Appl. Surf. Sci..

[27] Islam M.S., Deng Y., Tong L., Faisal S.N., Roy A.K., Minett A.I., Gomes V.G. (2016). Grafting carbon nanotubes directly onto carbon fibers for superior mechanical stability: Towards next generation aerospace composites and energy storage applications. Carbon.

[28] Wang L., Liu N., Guo Z., Wu D., Chen W., Chang Z., Yuan Q., Hui M., Wang J. (2016). Nitric Acid-Treated Carbon Fibers with Enhanced Hydrophilicity for Candida tropicalis Immobilization in Xylitol Fermentation. Materials.

[29] Wu T., Wang G., Dong Q., Qian B., Meng Y., Qiu J. (2016). Asymmetric capacitive deionization utilizing nitric acid treated activated carbon fiber as the cathode. Electrochim. Acta.

[B30-molecules-25-01295] 30.For comparison, TGA and Raman spectra of GO material and rGO aerogels have been recorded and analyzed in our previous work in ref [32].

[31] Ganguly A., Sharma S., Papakonstantinou P., Hamilton J. (2011). Probing the Thermal Deoxygenation of Graphene Oxide Using High-Resolution In Situ X-ray-Based Spectroscopies. J. Phys. Chem. C.

[32] Vrettos K., Karouta N., Loginos P., Donthula S., Gournis D., Georgakilas V. (2018). The Role of Diamines in the Formation of Graphene Aerogels. Front. Mater..

[33] Staudenmaier L. (1898). Verfahren zur Darstellung der Graphitsäure. Ber. Der Dtsch. Chem. Ges..

[34] Antonelou A., Sygellou L., Vrettos K., Georgakilas V., Yannopoulos S.N. (2018). Efficient defect healing and ultralow sheet resistance of laser-assisted reduced graphene oxide at ambient conditions. Carbon.

